# Nephrogenic Adenoma: A Pitfall on Frozen Section of Urethral Strictures

**DOI:** 10.1177/10668969241286069

**Published:** 2024-10-03

**Authors:** Alexander M. Oberc, Christopher Sherman, Michelle R. Downes

**Affiliations:** 1 7938 Department of Laboratory Medicine & Pathobiology, University of Toronto, Toronto, Canada; 2 Sunnybrook Health Sciences Centre, 7938 University of Toronto, Toronto, Canada

**Keywords:** urethral stricture, nephrogenic adenoma, nephrogenic metaplasia, frozen section

## Abstract

Urethral strictures are a common cause of urinary obstruction which can be treated with surgical resection. Frozen sections are rare and pose a diagnostic challenge to pathologists due to the presence of benign lesions such as nephrogenic adenoma. We retrospectively examined all specimens of urethral stricture resections submitted to pathology at our institution from 2012 to 2022 (n = 258). Final pathology reports were searched to identify patients with dysplasia, carcinoma, or nephrogenic adenoma. When available, frozen section reports were also examined and compared to the final report, and additional clinical history and microscopic images were collected for patients with nephrogenic adenoma. Nephrogenic adenoma was identified in 3.8% (10/258) of urethral stricture resections. Dysplasia was identified in one patient who underwent two separate resections, and squamous cell carcinoma was found in one resection. Intraoperative frozen section was requested in 3.4% of resections (9/258). In two resections, an initial diagnosis of squamous cell carcinoma was initially favoured, however when reviewed with a genitourinary pathologist the diagnosis was changed to “reactive process” with a final diagnosis of nephrogenic adenoma. Nephrogenic adenoma can be challenging on frozen section due to variable architectural patterns, inflammation, and reactive changes. While urethral strictures are relatively common, their assessment by frozen section is rare and pathologists may lack familiarity with the variable morphology of benign entities that can be seen on frozen section resulting in their misinterpretation. We highlight this potential diagnostic pitfall and demonstrate the value of a second opinion prior to definitive frozen section diagnosis of malignancy.

## Introduction

Urethral strictures are a common cause of urinary obstruction which occur predominantly in men.^1^ Urethral strictures result from subepithelial scarring which compresses the urethral lumen. While historically the main cause of urethral strictures was infection, more recently iatrogenic causes have become more common. Iatrogenic strictures can occur following treatment of many common urologic conditions such as prostate cancer, benign prostate hyperplasia, and urothelial cancer.^2,3^ The treatment of these conditions, which can involve instrumentation, transurethral ablation, surgical resection, or radiation, are all associated with a risk of developing urethral strictures.

While many patients with urethral strictures can be treated endoscopically, urethral resections (known as urethroplasty) may be indicated for strictures that are greater than 2 cm, have an obliterated lumen, or involve the posterior or pendulous urethra.^4^ Intraoperative frozen section may be requested during urethroplasty if there is a concern of malignancy, particularly in patients with a history of prostate or urothelial cancer. The evaluation of these specimens by pathologists can be challenging due to their rarity and the presence of benign conditions mimicking carcinoma such as nephrogenic adenoma.

Nephrogenic adenoma, also known as nephrogenic metaplasia, is a rare benign lesion thought to arise from implantation of renal tubular cells in the urinary tract.^5^ While nephrogenic adenoma most commonly occurs in the bladder, it can also be found in the urethra and is associated with many of the same risk factors as urethral strictures.^6^ Nephrogenic adenoma has several histologic patterns including tubular, vascular-like, corded, polypoid, papillary, and flat. Due to its variable morphology and infiltrative appearance, this benign lesion can mimic carcinoma and can be particularly challenging with suboptimal morphology during frozen section. Here we present a retrospective review of all urethral stricture resections at our institution, and we highlight two patients where nephrogenic adenoma was initially misinterpreted as carcinoma during frozen section.

## Methods

We retrospectively examined all pathology reports of urethral stricture resections submitted to Sunnybrook Health Sciences Centre, Toronto, Canada from 2012 to 2022 (n = 258). Final pathology reports were searched to identify specimens with dysplasia, carcinoma, or nephrogenic adenoma. When available, frozen section reports were also examined and compared to the final report. Additional clinical history and microscopic images were collected for patients with nephrogenic adenoma.

## Results

Nephrogenic adenoma was identified in the final report of 3.8% (10/258) of urethral stricture resections. Urothelial dysplasia was identified in one patient who underwent two separate urethral resections. One patient was found to have squamous cell carcinoma. Intraoperative frozen section was requested in 3.4% of all resections (9/258). We identified two difficult resections where nephrogenic adenoma was encountered at frozen section. Both patients had no prior history of urothelial dysplasia or carcinoma.

**Figure 1.**
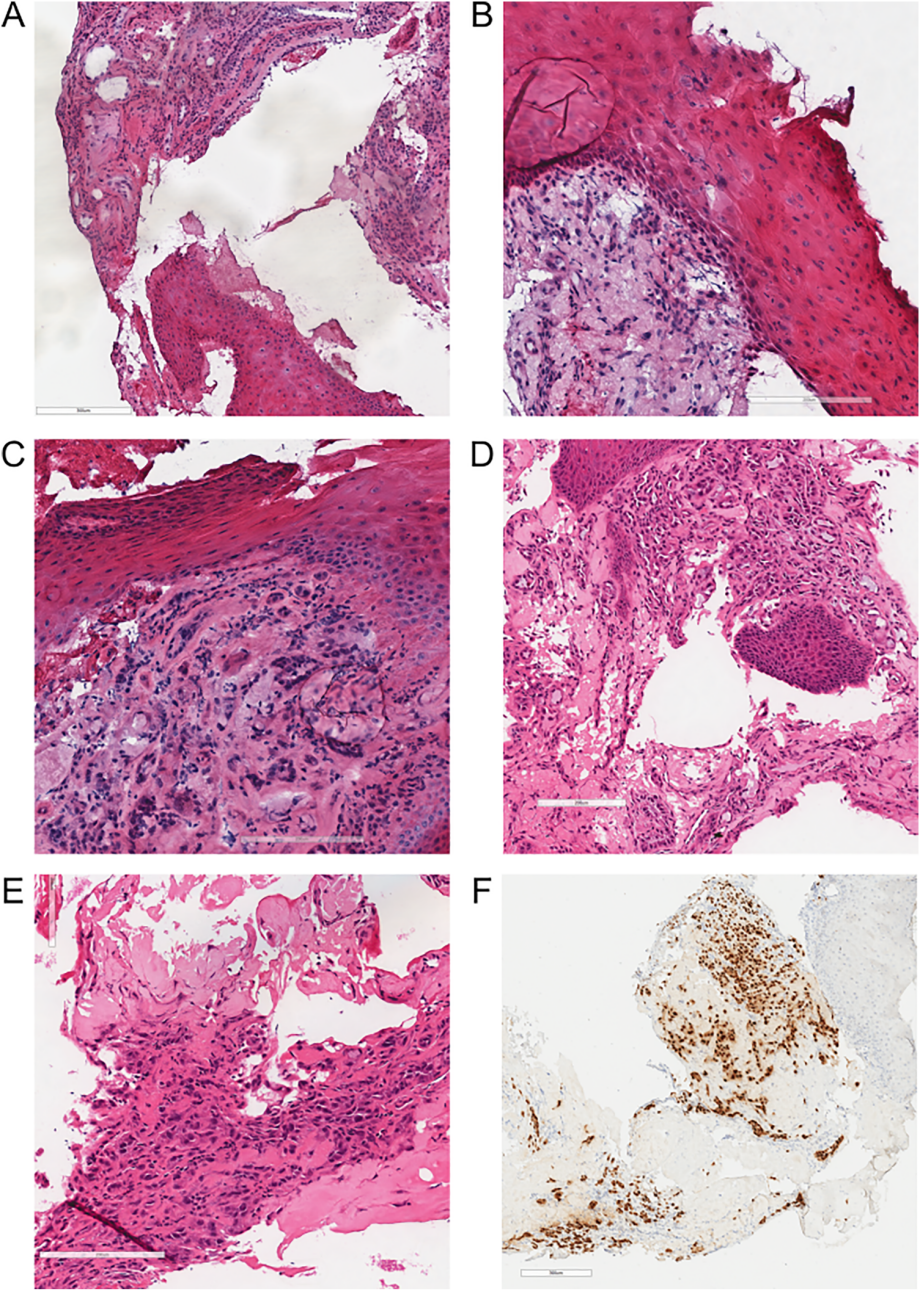
Representative frozen and paraffin histology sections of a urethral stricture resection with squamous metaplasia and nephrogenic adenoma described in patient 1. (A-C) Frozen H&E stained sections showing urethral mucosa with squamous metaplasia and an underlying tubular proliferation of nephrogenic adenoma within the lamina propria. (D-E) Paraffin H&E stained section showing glandular nephrogenic adenoma with a single layer lining with flattened to hobnailed morphology and luminal secretions. (F) PAX8 immunohistochemistry staining nuclei within glandular areas confirms the presence of nephrogenic adenoma.

The first patient was a man in his 20's with a history of recurrent urethral strictures who had undergone multiple previous dilations. The patient underwent a urethral reconstruction with a buccal graft and intraoperatively the surgeons found a discoloured focus of tissue within the stricture. A frozen section was requested with a question of malignancy (Figure 1).

**Figure 2.**
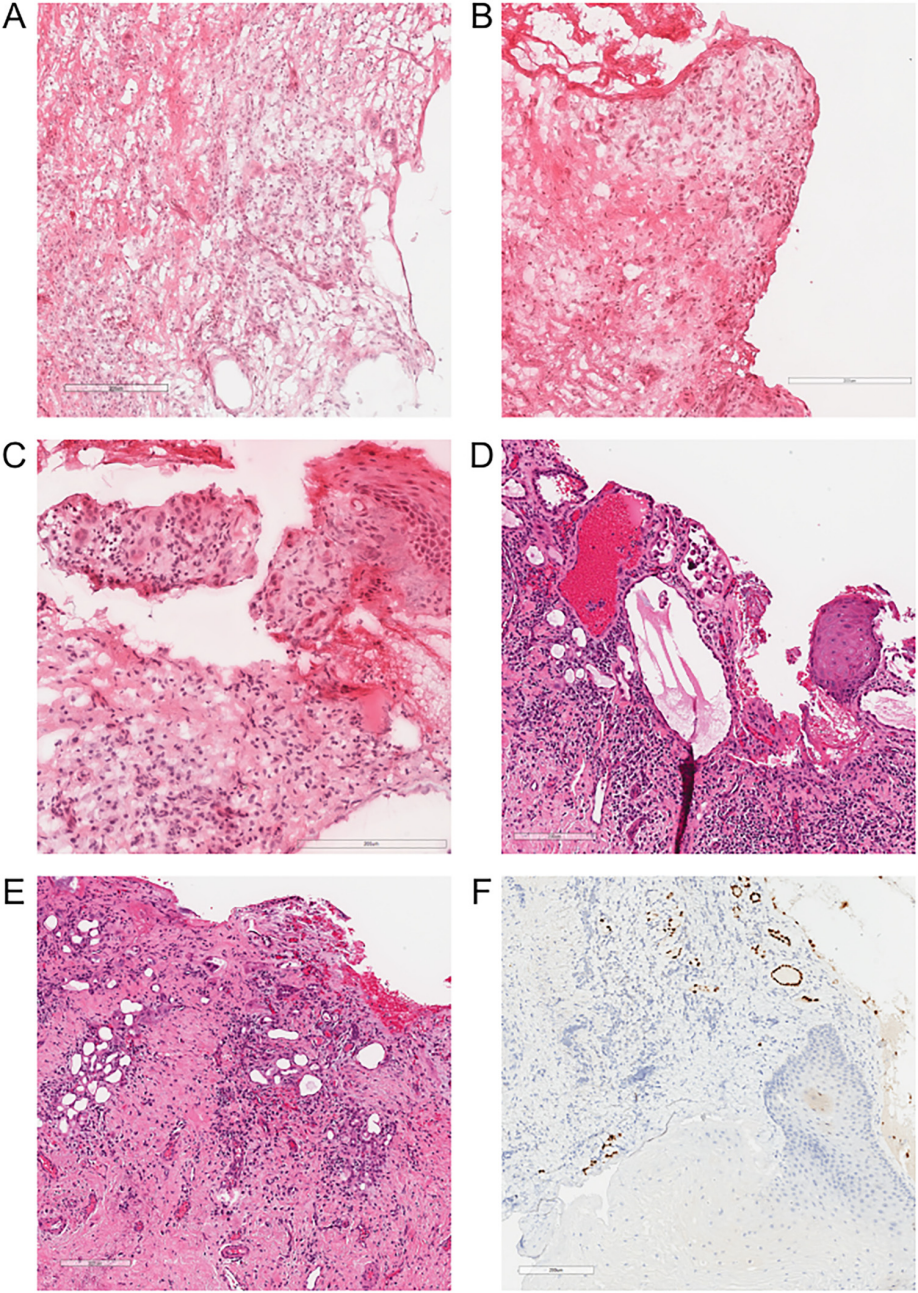
Representative frozen and paraffin histology sections of a urethral stricture resection with squamous metaplasia and nephrogenic adenoma described in patient 2. (A-C) Frozen H&E stained sections showing urethral mucosa with focal squamous epithelium, an ill-defined tubular proliferation within the lamina propria, and extensive artefactual changes. (D-E) Paraffin H&E stained section showing glandular elements with hobnailed and focal micropapillary morphologies. (F) PAX8 immunohistochemistry stains nuclei in glandular areas confirming the presence of nephrogenic adenoma.

The second patient was a man in his 60's with a long standing history of urethral strictures who had undergone multiple dilations and visual internal urethrotomies. A stricture resection and reconstruction with a buccal graft was performed. Intraoperatively, a nodule was noted proximal to the stricture which was suspected to be a fibroepithelial polyp or squamous cell carcinoma and a frozen section was requested (Figure 2).

Both patients’ resections showed squamous epithelium overlying infiltrative appearing nests and glands within a fibrotic and inflamed stroma. Initial diagnoses of squamous cell carcinoma were initially favoured by the intraoperative pathologists. When the slides were reviewed with a genitourinary pathologist, the diagnoses were changed to “reactive process”. On paraffin sections, the benign features of both the squamous epithelium and infiltrative glands were more evident. The squamous epithelium showed no significant atypia and did not infiltrate into the underlying stroma. The infiltrative nests similarly showed bland nuclear features and a hobnailed morphology with no significant mitotic activity. PAX8 immunohistochemistry was positive in the infiltrative glands leading to final diagnoses of nephrogenic adenoma and squamous metaplasia.

## Discussion

We performed a review of urethral stricture resections at our institution where we found a relatively low but clinically significant presence of nephrogenic adenoma. We reviewed two challenging resections where nephrogenic adenoma was encountered on intraoperative frozen section which were initially favoured to represent a malignant process. We highlighted this potential diagnostic pitfall at frozen section and the value of a second opinion prior to a definitive frozen section diagnosis of malignancy.

The rates of dysplasia and malignancy found in urethral stricture resections have not been reported previously. We found that both dysplasia and malignancy are relatively rare and are each found in less than 1% of all urethral stricture resections. Among all malignancies of the urinary tract, primary urethral carcinomas are relatively rare and they frequently show squamous differentiation.^7^ Nephrogenic adenoma was more common than malignant and dysplastic lesions and was found in 3.8% of urethral stricture resections. While nephrogenic adenoma is considered benign, there is a high rate of local recurrence following transurethral resections within the bladder^8^ and its long-term outcomes within the urethra have yet to be reported.

The challenges of diagnosing nephrogenic adenoma at frozen section have not been previously discussed in the literature. While squamous cell carcinoma was the main diagnostic consideration in both patients presented, several other malignant entities which involve the lower urinary tract should be considered in the differential of nephrogenic adenoma. A common malignant mimic of nephrogenic adenoma in the urethra of men is prostatic adenocarcinoma.^6,9^ Other known mimickers include urothelial carcinoma (particularly glandular, tubular, and microcystic variants) and clear cell adenocarcinoma.^10,11^ Several morphologic features can be used to differentiate nephrogenic adenoma from these malignant entities. Squamous carcinoma often shows keratinization within infiltrative nests, however in both patients keratinization was only found along the luminal surface. Compared to glandular carcinomas, in nephrogenic adenoma tubules are composed of a single layer of cells which typically have a hobnailed morphology.^11^ Nephrogenic adenoma should only show focal nuclear enlargement, focal prominent nucleoli, rare mitotic figures, and no desmoplasia. By immunohistochemistry, nephrogenic adenoma should stain positive for PAX8, PAX2, keratin 7, and keratin 34betaE12, and typically does not stain for NKX3.1, p63 or PSA.^12,13^ Notably, GATA3 is positive in 40% of specimens with nephrogenic adenoma and should be interpreted with caution when used to differentiate from urothelial carcinoma. However, as immunohistochemistry is not available during frozen sections it is important to recognise the features of nephrogenic adenoma on morphology alone.

While frozen sections can be valuable to surgeons when encountering unexpected lesions during urethral resections, our findings reveal several pitfalls. The presence of nephrogenic adenoma, squamous metaplasia, inflammation, and reactive nuclear atypia can lead to both the overdiagnosis and underdiagnosis of malignant conditions. Given the rarity of frozen sections during urethral resections, our findings emphasize the importance of pathologists to be familiar with these entities and to seek a second opinion when possible.

The Authors declare that there are no conflicts of interest.
